# Supplementary material for: Galectin-9 interacts with PD-1 and TIM-3 to regulate T cell death and is a target for cancer immunotherapy
Source: Nat Commun. 2021 Feb 5;12:832. doi: 10.1038/s41467-021-21099-2 (PMC7864927; doi:10.1038/s41467-021-21099-2)
Supplement: Supplementary file 1 — Supplementary Information [file 41467_2021_21099_MOESM1_ESM.pdf]

a

Uniprot ID	Protein Name	Gene Name	Control	PD-1.3F	PD-1.3F/ Control
Q9Y5B9	FACT complex subunit SPT16	SUPT16H	102	51983	507.44
**O00182**	**Galectin-9**	**LGALS9**	**2406**	**52710**	**21.90**
P84243	Histone H3.3	H3F3A	15477	133080	8.60
P05141	ADP/ATP translocase 2	SLC25A5	100828	374705	3.72
P18621	60S ribosomal protein L17	RPL17	20253	65915	3.25
O60825	6-phosphofructo-2-kinase	PFKFB2	43065	126575	2.94
Q15366	Poly(rC)-binding protein 2	PCBP2	23514	61835	2.63
O60234	Glia maturation factor gamma	GMFG	59795	144035	2.41
P16104	Histone H2AX	H2AFX	324660	748660	2.31
P11142	Heat shock cognate 71 kDa protein	HSPA8	180175	370465	2.06

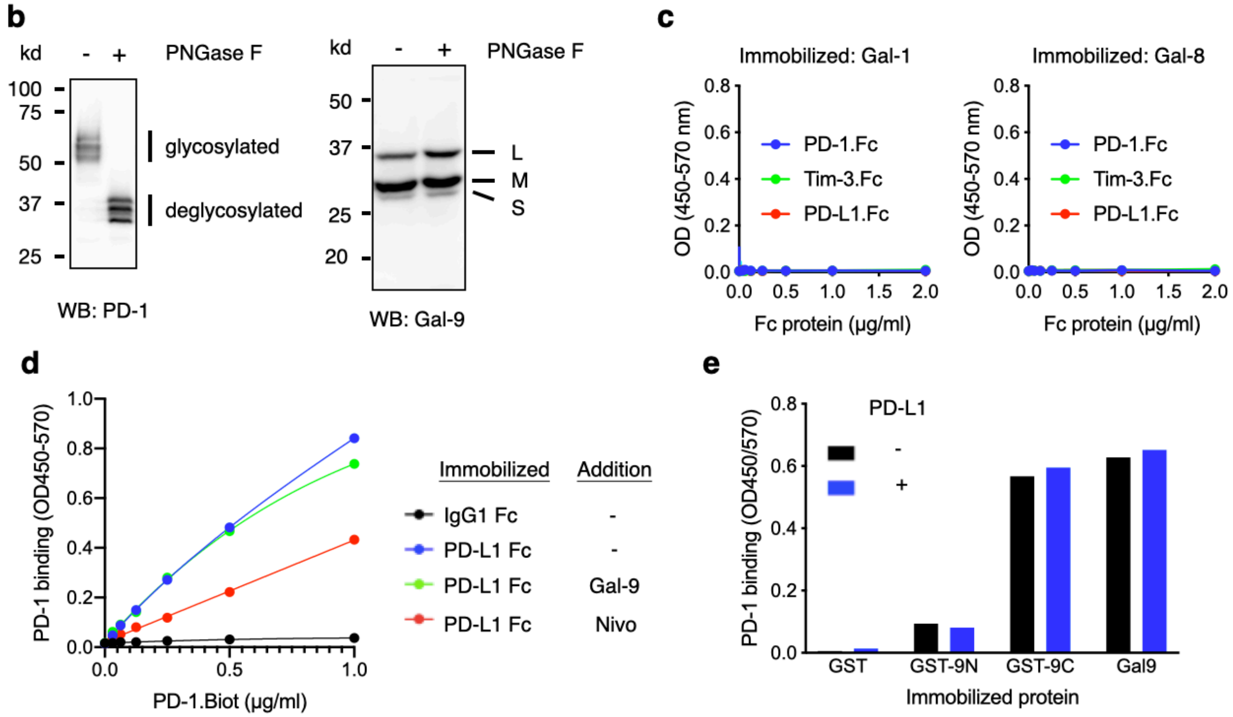

Supplementary Fig. 1. Galectin-9 is a PD-1-binding Protein. a, Putative PD-1-binding proteins identified by immunoprecipitation-mass spectrometry. **b**, PD-1-transduced Jurkat cell lysates were treated with or without peptide-N-glycosidase F, and subjected to Western blotting with PD-1 (left) or Gal-9 (right) antibodies. **c**, Binding of indicated Fc-fusion proteins to Gal-1 or Gal-8 coated on MaxiSorp plate. **d**, Binding of PD-1-biotin to indicated proteins immobilized on plate in the presence or absence of Gal-9 or Nivo. **e**, Binding of PD-1.Fc to indicated immobilized proteins in the absence or presence of PD-L1. Data are representative of three (**b**) or two (**c**-**e**) independent experiments. Source data are provided as a Source Data file.

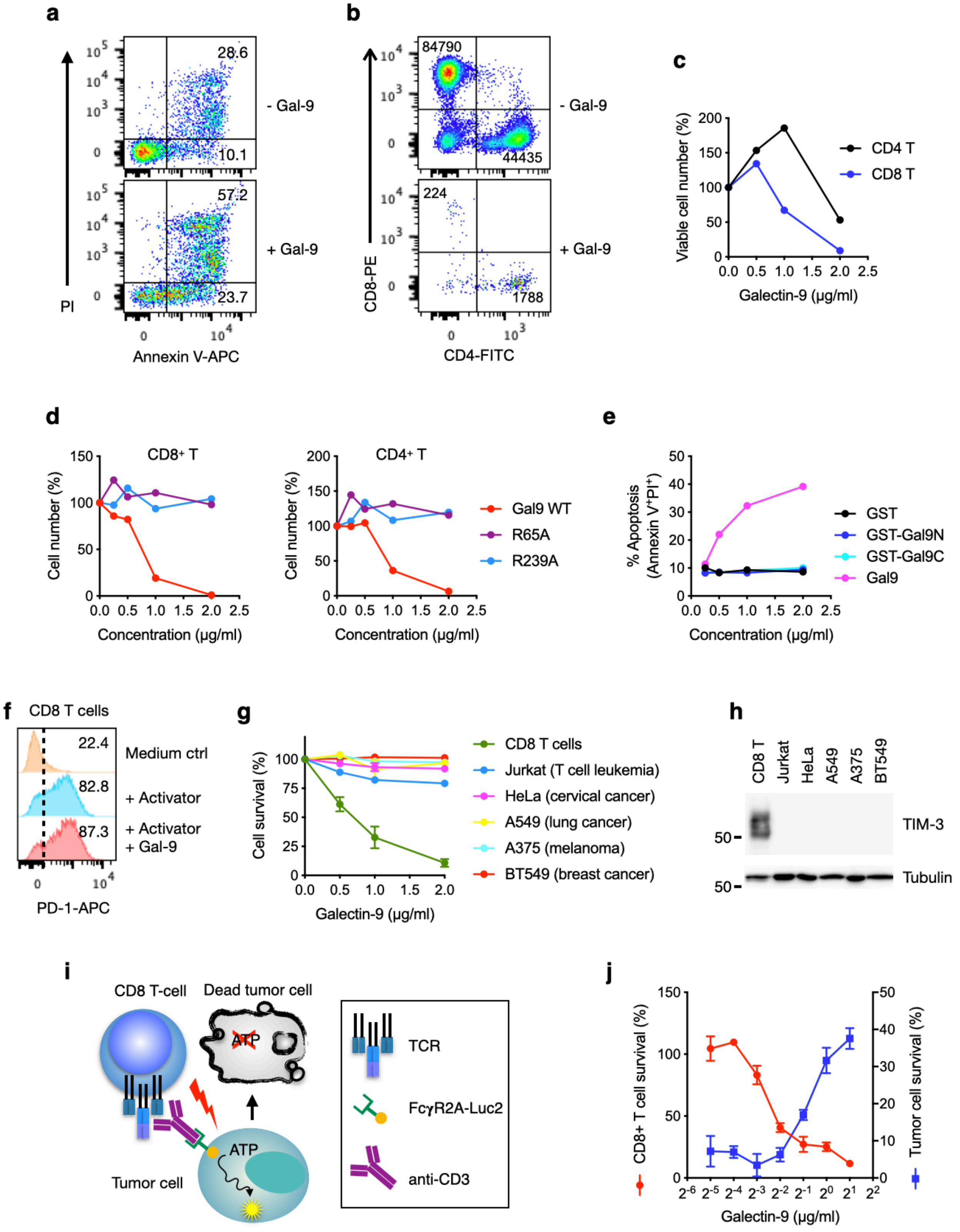
a
PI
Annexin V-APC
28.6
10.1
57.2
23.7
- Gal-9
+ Gal-9
b
CD8-PE
CD4-FITC
84790
44435
224
1788
- Gal-9
+ Gal-9
c
Viable cell number (%)
Galectin-9 (μg/ml)
CD4 T
CD8 T
d
CD8+ T
CD4+ T
Cell number (%)
Concentration (μg/ml)
Gal9 WT
R65A
R239A
e
% Apoptosis (Annexin V+PI+)
Concentration (μg/ml)
GST
GST-Gal9N
GST-Gal9C
Gal9
f
CD8 T cells
22.4
82.8
87.3
Medium ctrl
+ Activator
+ Activator + Gal-9
PD-1-APC
g
Cell survival (%)
Galectin-9 (μg/ml)
CD8 T cells
Jurkat (T cell leukemia)
HeLa (cervical cancer)
A549 (lung cancer)
A375 (melanoma)
BT549 (breast cancer)
h
CD8 T
Jurkat
HeLa
A549
A375
BT549
TIM-3
Tubulin
50
i
CD8 T-cell
Dead tumor cell
ATP
Tumor cell
TCR
FcγR2A-Luc2
anti-CD3
j
CD8+ T cell survival (%)
Tumor cell survival (%)
Galectin-9 (μg/ml)

Supplementary Fig. 2. Cell type-selective induction of apoptosis by Gal-9. **a,** Preactivated human T cells were incubated with or without 2 ug/ml Gal-9 for one day. Cell death was assessed using annexin V binding assay. Numbers indicate percentage of cells in quadrants. **b** and **c**, Preactivated human T cells were incubated with increasing concentrations of Gal-9 for one day and stained for CD4 and CD8. Viable cells were gated according to normal scatter (FSC/SSC) parameters and PI exclusion, and CD4 and CD8 T cells quantified using counting beads. Numbers indicate cell count in quadrant. Viable cell number in the absence of Gal-9 was taken as 100%. **d**, Survival of CD8 or CD4 T cells after treatment with wildtype Gal-9 or loss-of-function mutants of individual CRDs. **e**, Dose-dependent induction of CD8 T cell apoptosis by Gal-9 or GST-fusion proteins of its individual CRDs, as measured by annexin V-binding/PI staining assay. **f**. Flow cytometric analysis of PD-1 expression in CD8 T cells under indicated treatment conditions. Numbers denote percentage of PD-1^+ cells. Data are representative of three independent experiments. **g**, Differential sensitivity of various cell types to Gal-9-induced cell death. Cell survival was determined by MTS assay. OD values in the absence of Gal-9 was taken as 100%. Mean values +/- SEM of three independent experiments. **h**, Western blot showing expression of TIM-3 in cell types used in **g**. Data are representative of two independent experiments. **i**, A co-culture system of engineered T cell cytotoxicity toward tumor cells. Target cancer cells were engineered to express Fcγ receptor 2A fused with the luciferase Luc2 at the C-terminus, and then incubated with CD8 T cells and the CD3 antibody OKT3. After incubation, target cell survival was determined by luciferase assay. **j**, Correlation analysis of CD8 T cell and target cancer cell survival in the co-culture. Mean values +/- SEM of three independent experiments. Source data are provided as a Source Data file.

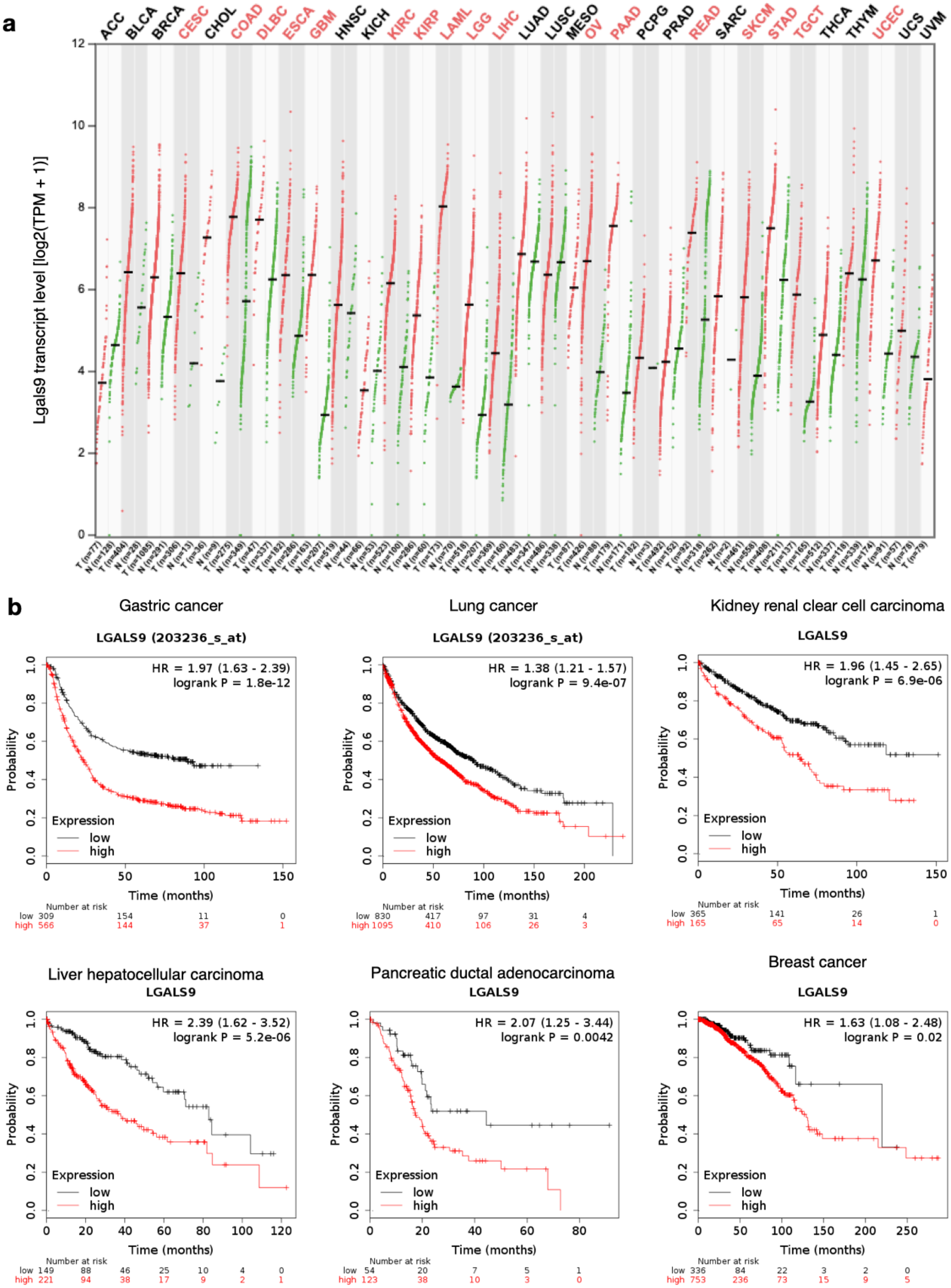
a
ACC BLCA BRCA CESC CHOL COAD DLBC ESCA GBM HNSC KICH KIRC KIRP LAML LGG LIHC LUAD LUSC MESO OV PAAD PCPG PRAD READ SARC SKCM STAD TGCT THCA THYM UCEC UCS UVM
Lgals9 transcript level [log2(TPM + 1)]
b
Gastric cancer
LGALS9 (203236_s_at)
HR = 1.97 (1.63 - 2.39)
logrank P = 1.8e-12
Lung cancer
LGALS9 (203236_s_at)
HR = 1.38 (1.21 - 1.57)
logrank P = 9.4e-07
Kidney renal clear cell carcinoma
LGALS9
HR = 1.96 (1.45 - 2.65)
logrank P = 6.9e-06
Liver hepatocellular carcinoma
LGALS9
HR = 2.39 (1.62 - 3.52)
logrank P = 5.2e-06
Pancreatic ductal adenocarcinoma
LGALS9
HR = 2.07 (1.25 - 3.44)
logrank P = 0.0042
Breast cancer
LGALS9
HR = 1.63 (1.08 - 2.48)
logrank P = 0.02
Probability
Time (months)
Expression
low
high
Number at risk

Supplementary Fig. 3. Gal-9 is a target for cancer immunotherapy. a, Dot plot showing Gal-9 gene expression profile across all tumor samples (T, red dots) and paired normal tissues (N, green dots) in TCGA. Names of cancer types with significantly altered Gal-9 expression are shown in red. Each dot represent expression of one sample. Line represents median expression. Lgals9 expression in normal (N) and tumor (T) samples was compared using unpaired two-tailed t-test. **b**, Kaplan-Meier survival estimated using KM-Potter showing the relationship between Gal-9 expression in human cancers and overall survival of patients. Log-rank (Mantel-Cox) test.

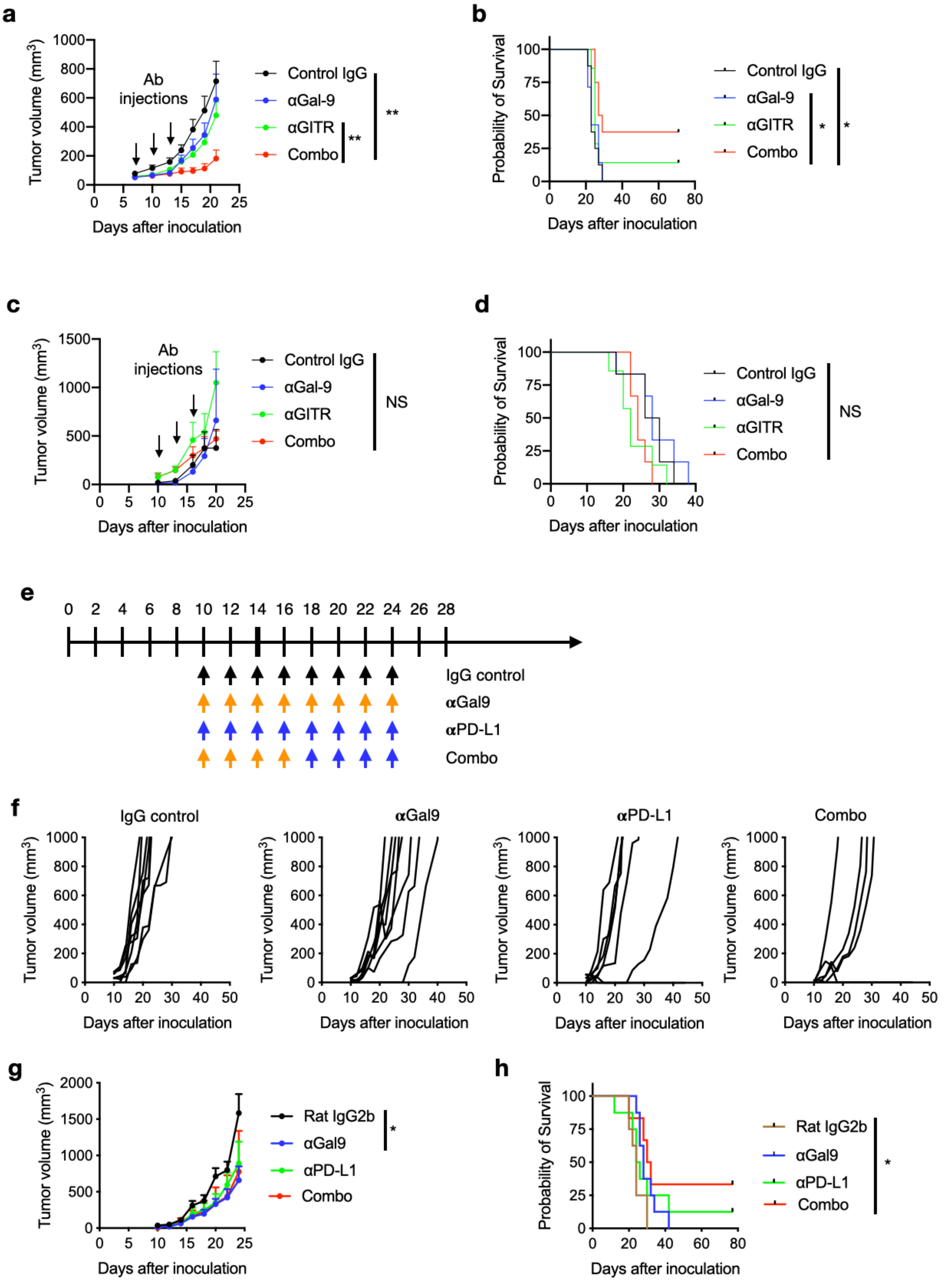
a
Tumor volume (mm3)
Ab injections
Days after inoculation
Control IgG
αGal-9
αGITR
Combo
**
**
b
Probability of Survival
Days after inoculation
Control IgG
αGal-9
αGITR
Combo
*
*
c
Tumor volume (mm3)
Ab injections
Days after inoculation
Control IgG
αGal-9
αGITR
Combo
NS
d
Probability of Survival
Days after inoculation
Control IgG
αGal-9
αGITR
Combo
NS
e
0 2 4 6 8 10 12 14 16 18 20 22 24 26 28
IgG control
αGal9
αPD-L1
Combo
f
IgG control
αGal9
αPD-L1
Combo
Tumor volume (mm3)
Days after inoculation
g
Tumor volume (mm3)
Days after inoculation
Rat IgG2b
αGal9
αPD-L1
Combo
*
h
Probability of Survival
Days after inoculation
Rat IgG2b
αGal9
αPD-L1
Combo
*

Supplementary Fig. 4. Effects of anti-Gal-9 and combination therapies in mouse tumor models. a, Average tumor growth of C57BL/6J mice inoculated with MC-38 mouse colon cancer cells and subjected to the indicated treatments. n=8 animals in control IgG, αGal-9, and Combo groups; n=7 animals in αGITR group. Control IgG vs combo, P=0.0023; αGITR vs combo, P=0.0087. **b.** Survival of C57BL/6J mice inoculated with MC-38 mouse colon cancer cells and subjected to the indicated treatments. Error bars represent SEM of the means. Statistical differences of tumor growth kinetics between treatment groups were assessed using unpaired two-tailed t-tests to compare area under the curves. Control IgG vs combo, P=0.0124; αGal-9 vs combo, P=0.0349. **c, d**, The average tumor growth and survival of C57BL/6J mice inoculated with B16F10 mouse melanoma cells and subjected to the indicated treatments. n=6 animals in control IgG, αGal-9, and Combo groups; n=7 animals in αGITR group. **e**-**h**, Response of BALB/cJ mice inoculated with EMT-6 mouse triple negative breast cancer cells to anti-Gal-9/anti-PD-L1 treatment. **e**, Treatment schedule. **f**. Individual tumor growth curves. **g**. Average tumor growth curves. n=8, 8, 8, 6 animals in control IgG, αGal-9, αPD-L1, and Combo group, respectively. Error bars represent SEM of the means. Statistical differences of tumor growth kinetics between treatment groups were assessed using unpaired two-tailed t-tests to compare area under the curves. Control IgG2b vs αGal-9, P=0.0166. **h**. Survival curves of mice in each treatment group. Log-rank (Mantel-Cox) tests were used for comparison of survival curves. Rat IgG2b vs combo, P=0.0374. Source data are provided as a Source Data file.

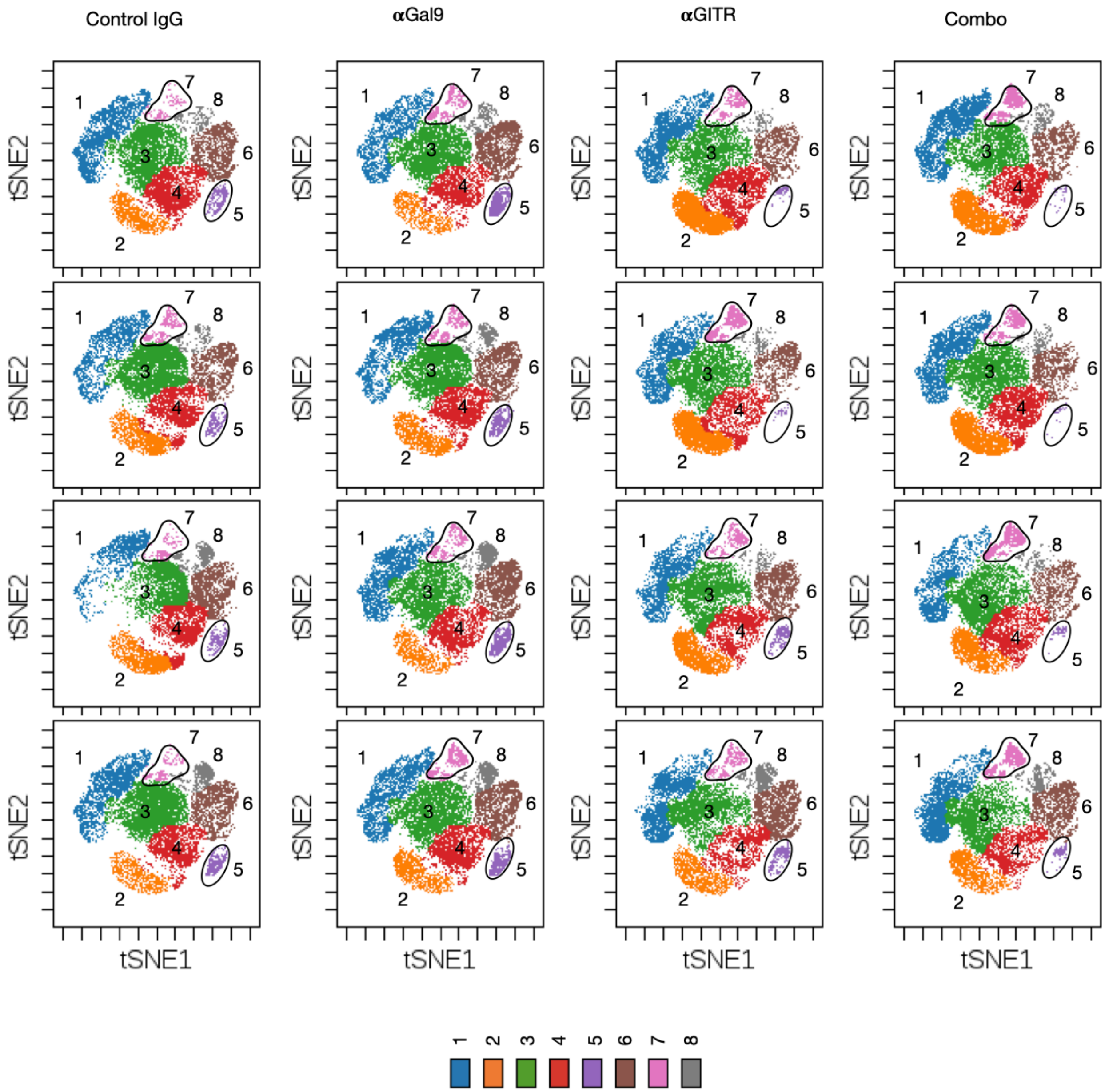

Supplementary Fig. 5. CyTOF analysis of tumor-infiltrating lymphocytes (TILs) after indicated treatments of mice with established MC-38 tumors. vi-SNE and FlowSOM analyses generated 8 color-coded clusters for TILs isolated from each sample (n =4 in each treatment group).

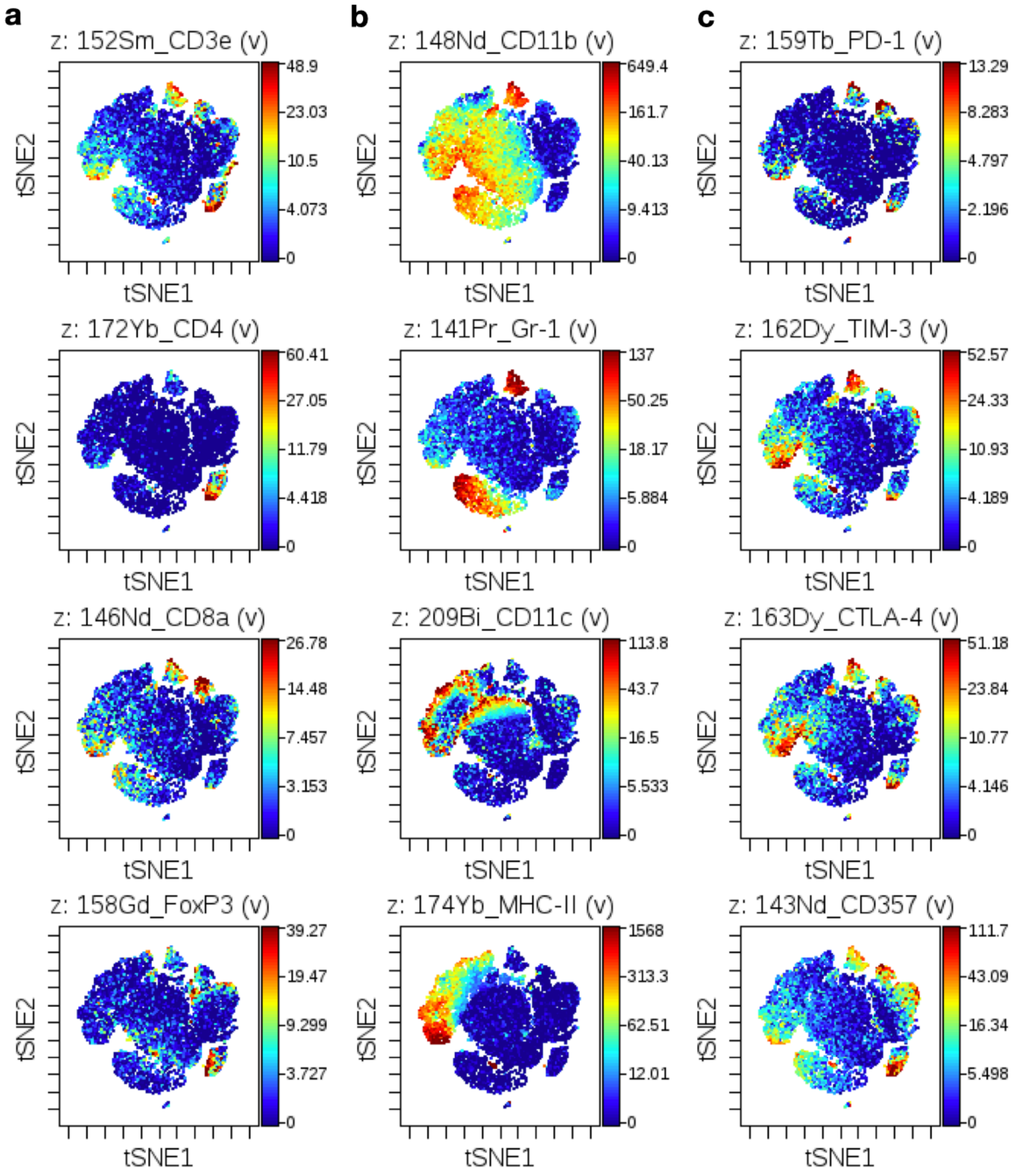

Supplementary Fig. 6. Marker expression in TIL clusters. a-c, t-SNE plot of TILs overlaid with the expression of selected markers for T cells (**a**), myeloid cells (**b**), and immune checkpoint molecules (**c**), in a sample from a representative mouse of the anti-Gal-9 treatment group.

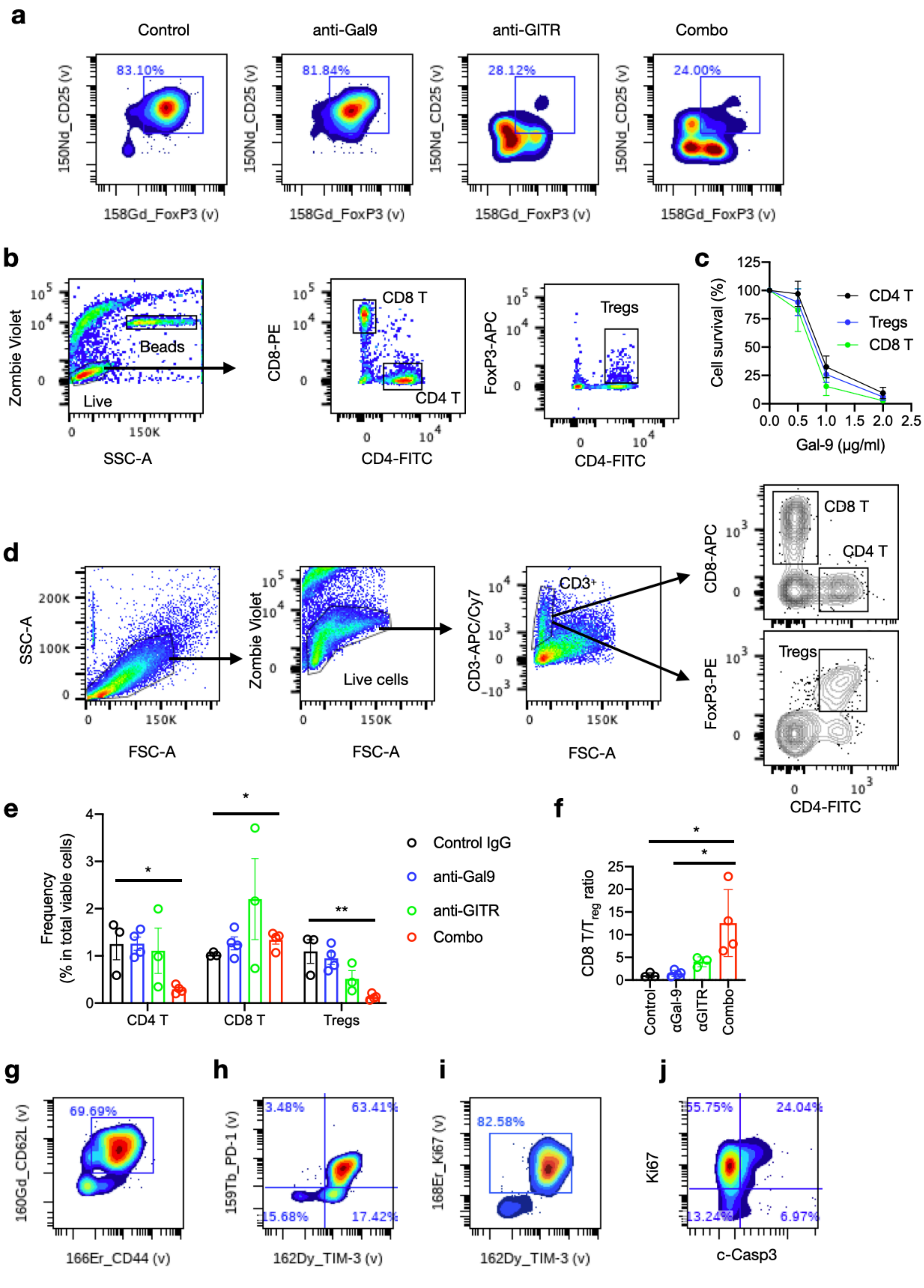
a
Control
anti-Gal9
anti-GITR
Combo
83.10%
81.84%
28.12%
24.00%
150Nd_CD25 (v)
158Gd_FoxP3 (v)
b
Zombie Violet
Beads
Live
SSC-A
CD8-PE
CD8 T
CD4 T
CD4-FITC
FoxP3-APC
Tregs
c
Cell survival (%)
CD4 T
Tregs
CD8 T
Gal-9 (µg/ml)
d
SSC-A
FSC-A
Zombie Violet
Live cells
CD3-APC/Cy7
CD3+
CD8-APC
FoxP3-PE
e
Frequency (% in total viable cells)
Control IgG
anti-Gal9
anti-GITR
Combo
CD4 T
CD8 T
Tregs
f
CD8 T/Treg ratio
Control
αGal-9
αGITR
Combo
g
69.69%
160Gd_CD62L (v)
166Er_CD44 (v)
h
3.48%
63.41%
15.68%
17.42%
159Tb_PD-1 (v)
162Dy_TIM-3 (v)
i
82.58%
168Er_Ki67 (v)
162Dy_TIM-3 (v)
j
55.75%
24.04%
13.24%
6.97%
Ki67
c-Casp3

Supplementary Fig. 7. Characterization of CD8 T cells and T_{reg} cells that responded to anti-Gal-9 antibody treatment. a. Density plots of CyTOF data showing enrichment of Tregs ($CD25^{+}FoxP3^{+}$) in MC-38 TIL CD4 T cells in control and anti-Gal-9 treated mice but not in anti-GITR or Combo treated mice. **b** and **c**, Activated human T cells were treated with or without Gal-9 and then subjected to flow cytometric analysis of viable cell counts with counting beads. **b**, Gating strategy. **c**. Survival of T cell subsets after Gal-9 treatment. n = 3 independent experiments. **d**-**f**, Determination of T cell frequency and CD8 T cell/Treg ratio in MC-38 TILs after indicated treatments by flow cytometry. **d**, Gating strategy. **e**, Frequency of CD4 T cells, CD8 T cells, and T_{reg} cells. n = 3, 4, 3, and 4 mice in control IgG, αGal-9, αGITR, and combo groups, respectively. CD4 T cells, control IgG2b vs combo, P=0.0196; CD8 T cells, control IgG2b vs combo, P=0.0380; T_{reg} cells, control IgG2b vs combo, P=0.0064. **f**, CD8 T/T_{reg} ratios in TILs from the 4 treatment groups. n = 3, 4, 3, and 4 mice in control IgG, αGal-9, αGITR, and combo groups, respectively. Control IgG2b vs combo, P=0.0469; αGal-9 vs combo, P=0.0242. **g**-**j,** Central memory (**g**), exhaustion (**h**), and proliferation (**i, j**) marker expression in anti-Gal-9-expanded CD8 T_1 subset. Unpaired two-tailed t tests were used for comparing means between treatment groups. Each circle represents one mouse. Error bars represent SEM. Source data are provided as a Source Data file.

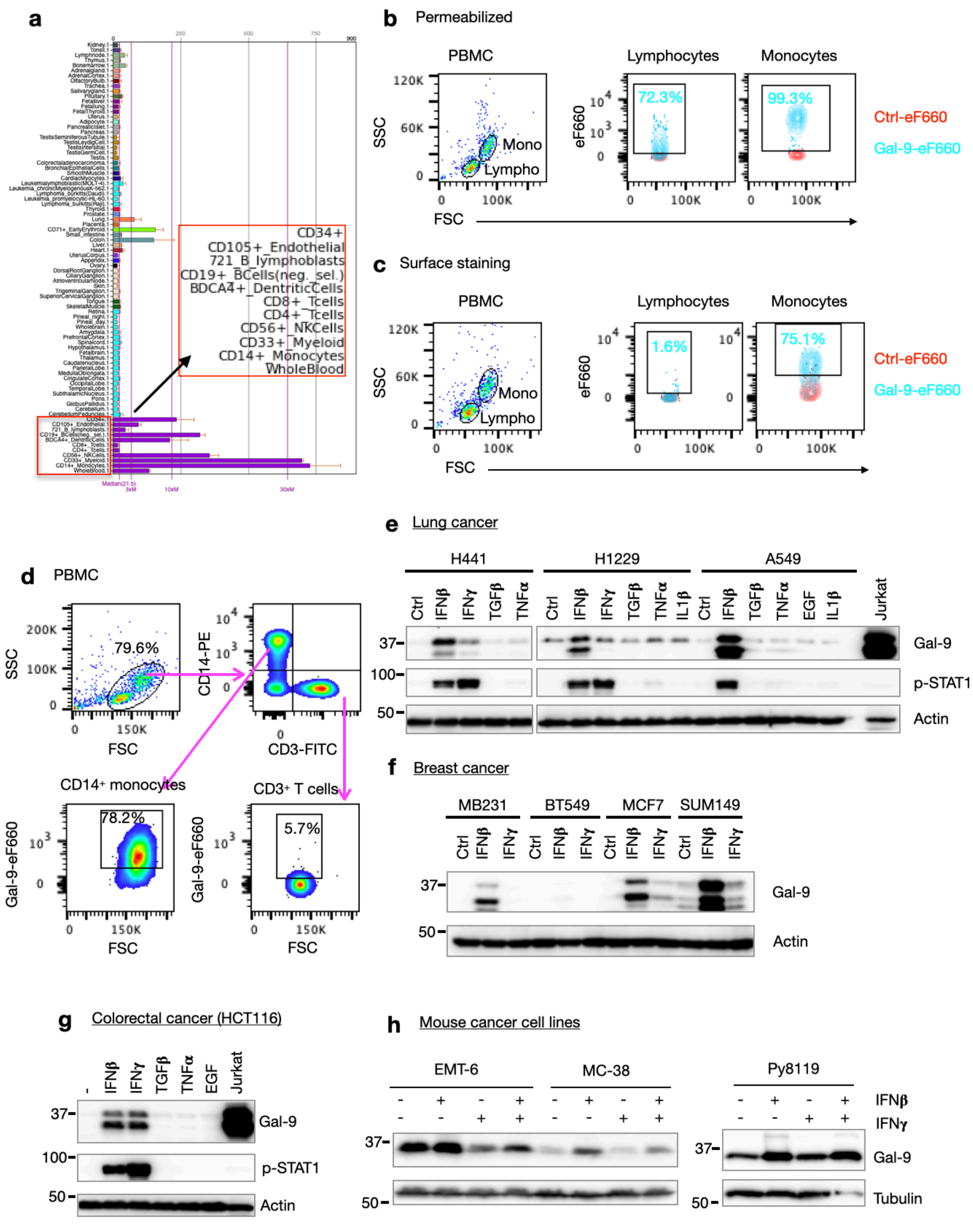
a
CD34+
CD105+_Endothelial
721_B_lymphoblasts
CD19+_BCells(neg._sel.)
BDCA4+_DentriticCells
CD8+_Tcells
CD4+_Tcells
CD56+_NKCells
CD33+_Myeloid
CD14+_Monocytes
WholeBlood
b
Permeabilized
PBMC
Lymphocytes
Monocytes
SSC
FSC
Mono
Lympho
eF660
72.3%
99.3%
Ctrl-eF660
Gal-9-eF660
c
Surface staining
PBMC
Lymphocytes
Monocytes
1.6%
75.1%
Ctrl-eF660
Gal-9-eF660
d
PBMC
79.6%
CD14-PE
CD3-FITC
CD14+ monocytes
CD3+ T cells
78.2%
5.7%
Gal-9-eF660
e
Lung cancer
H441
H1229
A549
Ctrl
IFNβ
IFNγ
TGFβ
TNFα
IL1β
EGF
Jurkat
Gal-9
p-STAT1
Actin
f
Breast cancer
MB231
BT549
MCF7
SUM149
Gal-9
Actin
g
Colorectal cancer (HCT116)
Gal-9
p-STAT1
Actin
h
Mouse cancer cell lines
EMT-6
MC-38
Py8119
IFNβ
IFNγ
Gal-9
Tubulin

Supplementary Fig. 8. Regulation of Gal9 expression. a, High-density oligonucleotide array analysis of a panel of 79 human tissues/cell types showing that Gal-9 is preferentially expressed by immune cells (purple. See inset for cell types), especially antigen presenting cells. (data source: BioGPS). **b** and **c**, Flow cytometric analysis of permeabilized (**b**) and nonpermeabilized (**c**) PBMCs for detection of total (**b**) and surface (**c**) Gal-9 in lymphocytes and monocytes. **d**, Flow cytometric analysis of nonpermeabilized PBMCs for detection of surface Gal-9 on $CD3^+$ T cells and $CD14^+$ monocytes. **e-h,** Expression of Gal-9 protein after indicated treatment of representative cancer cell lines from humans (**e-g**) and mice (**h**). Experiments were repeated twice with similar results. Source data are provided as a Source Data file.

a

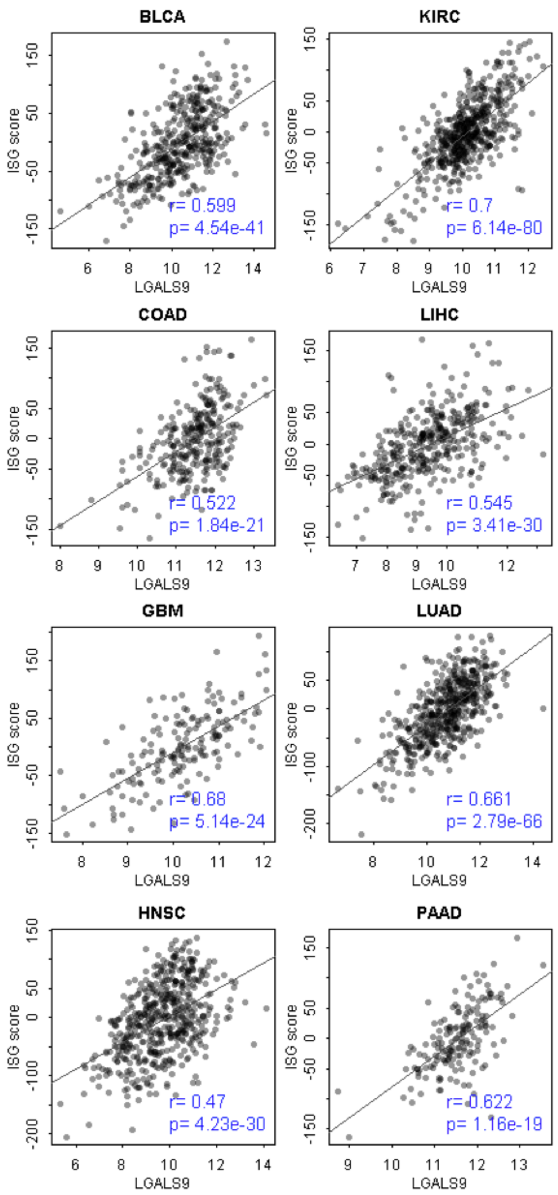

b

TCGA	Gal9_ISG_corr	CCLE	Gal9_ISG_corr
ACC	0.614		
BLCA	0.599	bladder	0.377
BRCA	0.795	breast	0.708
CESC	0.497		
COAD	0.522	colon	0.592
ESCA	0.484	esophagus	0.317
GBM	0.680	brain	0.711
LGG	0.737		
HNSC	0.470	head_neck	0.565
KICH	0.824		
KIRC	0.700		
KIRP	0.656	kidney	0.623
LAML	0.398	blood	0.427
DLBC	0.504		
LIHC	0.545	liver	0.726
LUAD	0.661	lung	0.572
LUSC	0.606		
MESO	0.644		
OV	0.759	ovary	0.660
PAAD	0.622	pancreas	0.686
PCPG	0.672		
PRAD	0.707		
SARC	0.743	bone	0.299
SKCM	0.721	skin	0.467
UVM	0.749		
STAD	0.642	stomach	0.435
TGCT	0.878		
THCA	0.826	thyroid	0.697
THYM	0.336		
UCEC	0.716	endometrium	0.678
UCS	0.830		

Supplementary Fig. 9. Co-expression of *LGALS9* with interferon-stimulated gens (ISGs) in multiple cancer types. a, Scatter plots showing co-expression of *LGALS9* with ISGs in multiple cancer types in TCGA. Two-tailed Pearson's correlation test. **b**, Pearson correlation coefficients of *LGALS9* and ISG expression in multiple cancer types in TCGA and corresponding cell lines in CCLE.

Supplementary Table 1. CyTOF antibody list

Target	Label	Clone	Specificity	Source	Catalog #
Ly-6G/Ly-6C, Gr-1	141Pr	RB6-8C5	Mouse	BioLegend	108402
KLRG1	142Nd	2F1	Mouse	BD	562190
CD357, GITR	143Nd	DTA1	Mouse	DVS-Fluidigm	3143019B
CD69	145Nd	H1.2F3	Mouse	BioLegend	104533
CD8a	146Nd	53-6.7	Mouse	DVS-Fluidigm	3146003B
CD223, LAG-3	147Sm	C9B7W	Mouse	BioLegend	125202
CD11b	148Nd	M1/70	Mouse	DVS-Fluidigm	3148003B
CD19	149Sm	6D5	Mouse	DVS-Fluidigm	3149002B
CD25	150Nd	3C7	Mouse	BioLegend	101902
TIGiT, Vstm3	151Eu	1G9	Mouse	BioLegend	142102
CD3, CD3e	152Sm	145-2C11	Mouse	BioLegend	100302
Caspase 3, cleaved	153Eu	D3E9	Human, Mouse	CST	9579BF
CD274, PD-L1	154Sm	10F.9G2	Mouse	BioLegend	124303
Foxp3	158Gd	FJK-16s	Mouse, Rat	DVS-Fluidigm	3158003A
CD279, PD-1	159Tb	29F.1A12	Mouse	BioLegend	135202
CD62L	160Gd	MEL-14	Mouse	DVS-Fluidigm	3160008B
T-bet	161Dy	4B10	Human, Mouse	DVS-Fluidigm	3161014B
TIM3, CD366	162Dy	RMT3-23	Mouse	DVS-Fluidigm	3162029B
CD152, CTLA-4	163Dy	9H10	Mouse	BioLegend	106202
Ly-6A/E, Sca-1	164Dy	D7	Mouse	DVS-Fluidigm	3164005B
CD44	166Er	IM7	Human, Mouse	BioLegend	103002
Ki67	168Er	B56	Mouse, Human	BD	556003
CD14	169Tm	Sa14-2	Mouse	BioLegend	123302
CD127, IL-7Ra	170Er	A7R34	Mouse	BioLegend	135029
CD4	172Yb	RM4-5	Mouse	BioLegend	100506
I-A/I-E MHC-II	174Yb	M5/114.15.2	Mouse	DVS-Fluidigm	3174003B
CD38	175Lu	90	Mouse	DVS-Fluidigm	3175014B
CD278, ICOS	176Yb	7E.17G9	Mouse	DVS-Fluidigm	3176014B
CD11c	209Bi	N418	Mouse	DVS-Fluidigm	3209005B
CD45	89Y	30-F11	Mouse	DVS-Fluidigm	3089005B

Supplementary Table 2. Other antibodies used in the study

REAGENT or RESOURCE	Supplier	Catalog #	Used at
FITC anti-human CD4 Antibody, clone RPA-T4	BioLegend	300506	1:20
PE anti-human CD8a Antibody, clone HIT8a	BioLegend	300908	1:20
APC anti-human CD279 (PD-1) Antibody, clone EH12.2H7	BioLegend	329908	1:50
PE anti-human CD366 (Tim-3) Antibody, clone F38-2E2	BioLegend	345006	1:50
eFluor 660 anti-human Galectin 9 Monoclonal Antibody, clone 9M1-3	Thermo Fisher Scientific	50-9116-42	1:20
FITC anti-human CD3 Antibody, clone HIT3a	BioLegend	300306	1:20
PE anti-human CD14 Antibody, clone 63D3	BioLegend	367104	1:20
FoxP3 Antibody, anti-human, APC, REAfinity	Miltenyi Biotec	130-125-580	1:50
Anti-mouse CD3-APC/Cy7	BioLegend	100221	1:20
Anti-mouse CD8-APC	BioLegend	100711	1:20
Anti-mouse CD4-FITC	BD Biosciences	553046	1:200
Anti-mouse FoxP3-PE	Invitrogen	12-5773-80	1:20
LEAF™ Purified anti-human CD3 antibody, clone OKT3	BioLegend	317304	Specified in Methods
LEAF™ Purified anti-human CD28 antibody, clone CD28.2	BioLegend	302914	Specified in Methods
Purified anti-mouse Galectin-9 Antibody, clone 108A2	BioLegend	137901	0.5 μg/ml
Purified Rat Anti-Mouse CD16/CD32 (Mouse BD Fc Block™), Clone 2.4G2	BD Biosciences	553142	1:50
Human TruStain FcX™ (Fc Receptor Blocking Solution)	BioLegend	422302	1:20
PD-1 (D4W2J) XP® Rabbit mAb	Cell Signaling Technology	86163	1:1000
Mouse anti-GALECTIN-9 Antibody, clone OTI1G3	Bio-Rad	VMA00212	1:1000
Peroxidase AffiniPure Goat Anti-Human IgG, Fcγ fragment specific	Jackson ImmunoResearch Inc.	109-035-098	1:10000
Peroxidase-AffiniPure Goat Anti-Rabbit IgG (H+L) antibody	Jackson ImmunoResearch Inc.	111-035-003	1:10000
Peroxidase-AffiniPure Goat Anti-Mouse IgG (H + L) antibody	Jackson ImmunoResearch Inc.	115-035-003	1:10000
Anti-mouse Galectin-9 antibody InVivoMAb, clone RG9-1	Bio X Cell	BE0218	100 μg/mouse
Anti-TIM-3 Rabbit antibody	R&D Systems	MAB23652	2 μg/ml
Anti-mouse GITR antibody InVivoMab, clone DTA-1	Bio X Cell	BE0063	100 μg/mouse
Rabbit Anti-Stat1, phospho (Tyr701) Monoclonal Antibody, Unconjugated, Clone 58D6	Cell Signaling Technology	9167	1:1000
Rat IgG2b isotype control antibody InVivoMab	Bio X Cell	BE0090	100 μg/mouse
InVivoMAb anti-mouse PD-L1 (B7-H1)	Bio X Cell	BE0101	100 μg/mouse
Mouse IgG1, kappa Isotype Ctrl PE	BioLegend	400112	1:20
Mouse IgG1, κ Isotype Ctrl Antibody APC	BioLegend	400120	1:20

Supplementary Table 3. List of primers

Name	Sequence
Primers for mutagenesis	
Gal-9(R65A)_F	CTTCAACCCTgccTTTGAAGATGGAGGG
Gal-9(R65A)_R	TGGAAGGCAATGTCATTTC
Gal-9(R239A)_F	CCTGAACCCCgccTTTGATGAGAATGCTG
Gal-9(R239A)_R	TGGAAGGCGATGTGGTTC
PD-1(N49Q)_F	CGAAGGGGACcagGCCACCTTCAC
PD-1(N49Q)_R	GTCACCACGAGCAGGGCT
PD-1(N58Q)_F	CAGCTTCTCCcagACATCGGAGAG
PD-1(N58Q)_R	CAGGTGAAGGTGGCGTTG
PD-1(N74Q)_F	GAGCCCCAGCcagCAGACGGACA
PD-1(N74Q)_R	ATGCGGTACCAGTTTAGCAC
PD-1(N116Q)_F	GGCCCGGCGCcagGACAGCGGCA
PD-1(N116Q)_R	CTGACCACGCTCATGTGGAAGTCACGC
Primers for real-time PCR	
QPCR Gal-9_F	TCTGGGACTATTCAAGGAGGTC
QPCR Gal-9_R	CCATCTTCAAACCGAGGGTTG
QPCR GAPDH_F	GGAGCGAGATCCCTCCAAAAT
QPCR GAPDH_R	GGCTGTTGTCATACTTCTCATGG
Primers for recombinant protein expression	
EcoRI_Gal9_F	GAATTCatggccttcagcggttcc
XhoI_Gal9_R	gCTCGAGctatgtctgcacatgggt
XhoI_Gal9N_R	gCTCGAGctactggaagctgatgtaggacagc
EcoRI_Gal9C_F	GAATTCttcatcaccaccattctggg
NdeI_Leg9_F	gCATATGgccttcagcggttcccagg
XhoI_Leg9_R	gCTCGAGctatgtctgcacatgggtcagc
XhoI_Leg9N_R	gCTCGAGctactggaagctgatgtaggacagc
NdeI_Leg9C_F	gCATATGttcatcaccaccattctggg
