# Supplementary material for: Galectin-9 interacts with PD-1 and TIM-3 to regulate T cell death and is a target for cancer immunotherapy
Source: Nat Commun. 2021 Feb 5;12:832. doi: 10.1038/s41467-021-21099-2 (PMC7864927; doi:10.1038/s41467-021-21099-2)
Supplement: Supplementary file 3 — Reporting Summary [file 41467_2021_21099_MOESM3_ESM.pdf]

nature research

Corresponding author(s): Mien-Chie Hung
Riyao Yang

Last updated by author(s): 12/30/2020

Reporting Summary

Nature Research wishes to improve the reproducibility of the work that we publish. This form provides structure for consistency and transparency in reporting. For further information on Nature Research policies, see Authors & Referees and the Editorial Policy Checklist .

Statistics

For all statistical analyses, confirm that the following items are present in the figure legend, table legend, main text, or Methods section.

n/a	Confirmed	
[ ]	[x]	The exact sample size (*n*) for each experimental group/condition, given as a discrete number and unit of measurement
[ ]	[x]	A statement on whether measurements were taken from distinct samples or whether the same sample was measured repeatedly
[ ]	[x]	The statistical test(s) used AND whether they are one- or two-sided<br>*Only common tests should be described solely by name; describe more complex techniques in the Methods section.*
[x]	[ ]	A description of all covariates tested
[ ]	[x]	A description of any assumptions or corrections, such as tests of normality and adjustment for multiple comparisons
[ ]	[x]	A full description of the statistical parameters including central tendency (e.g. means) or other basic estimates (e.g. regression coefficient) AND variation (e.g. standard deviation) or associated estimates of uncertainty (e.g. confidence intervals)
[ ]	[x]	For null hypothesis testing, the test statistic (e.g. *F*, *t*, *r*) with confidence intervals, effect sizes, degrees of freedom and *P* value noted<br>*Give P values as exact values whenever suitable.*
[x]	[ ]	For Bayesian analysis, information on the choice of priors and Markov chain Monte Carlo settings
[x]	[ ]	For hierarchical and complex designs, identification of the appropriate level for tests and full reporting of outcomes
[ ]	[x]	Estimates of effect sizes (e.g. Cohen's *d*, Pearson's *r*), indicating how they were calculated

Our web collection on statistics for biologists contains articles on many of the points above.

Software and code

Policy information about availability of computer code

Data collection	Flow cytometry data were acquired using BD FACSDiva 8.0.1 (BD Biosciences). Western blotting data were collected using ImageQuant LAS 4000 (GE Healthcare Life Sciences). Protein binding assay and ELISA data were collected using Gen5 (BioTek). Real-time PCR was performed using the Applied Biosystem 7500 system. Primers for mutagenesis was designed using NEBaseChanger (http://nebasechanger.neb.com).
Data analysis	Flow cytometry data were analyzed using FlowJo 10.6.2 (BD). Western blotting and Duolink data were processed and analyzed using ImageJ 2.0.0-rc-69/1.52p (NIH). Statistical tests were run using Prism 8.4.1 (GraphPad). CyTOF data were analyzed using Cytobank (Cytobank Inc.). Single-cell RNA-seq data of human melanoma TILs (GSE120575) were reanalyzed with BBrowser2 (BioTuring). The heatmaps that show the expression of the indicated markers were generated using pheatmap R package version 1.0.12.

For manuscripts utilizing custom algorithms or software that are central to the research but not yet described in published literature, software must be made available to editors/reviewers. We strongly encourage code deposition in a community repository (e.g. GitHub). See the Nature Research guidelines for submitting code & software for further information.

Data

Policy information about availability of data

All manuscripts must include a data availability statement. This statement should provide the following information, where applicable:

- Accession codes, unique identifiers, or web links for publicly available datasets
- A list of figures that have associated raw data
- A description of any restrictions on data availability

Raw CyTOF data supporting the findings of this study have been deposited in FlowRepository (http://flowrepository.org/id/FR-FCM-Z2MJ) under the FlowRepository identifier FR-FCM-Z2MJ. Databases used in this study include The Cancer Genome Atlas (TCGA) (https://tcga-data.nci.nih.gov/tcga/), the Molecular Signatures Database (https://www.gsea-msigdb.org/gsea/ msigdb), and the National Center for Biotechnology Information Gene Expression Omnibus (NCBI-GEO) (https://www.ncbi.nlm.nih.gov/geo/query/acc.cgi?acc=GSE120575). The remaining data are available within the Article, Supplementary Information or available from the authors upon request. Source data are provided with this paper.

Field-specific reporting

Please select the one below that is the best fit for your research. If you are not sure, read the appropriate sections before making your selection.

- [x] Life sciences
- [ ] Behavioural & social sciences
- [ ] Ecological, evolutionary & environmental sciences

For a reference copy of the document with all sections, see nature.com/documents/nr-reporting-summary-flat.pdf

Life sciences study design

All studies must disclose on these points even when the disclosure is negative.

Sample size	Group sizes for in vivo validation experiments were selected empirically based upon prior knowledge of the intragroup variation of tumor challenges and immunotherapy treatment. Similarly, group sizes for in vitro experiments were also selected on the basis of prior knowledge of variation. Source of such prior knowledge include papers published by this and other groups (e.g.: Li et al. 2016 PMID: 27572267, Cha et al. 2018 PMID: 30118680). No sample size calculation was done as sample size selection with the above methods is sufficient to detect meaningful biological differences with good reproducibility.
Data exclusions	No data were excluded from analyses
Replication	Replicates were used in experiments as noted in figures. All attempts at replication were successful.
Randomization	Age and sex-matched animals were used for each experiment. Mice were randomized prior to tumor size measurement and antibody treatment. For experiments other than mice studies, samples were also randomly allocated into experimental groups.
Blinding	Blinding was not performed in mouse experiments because investigator needed to know the treatment groups in order to perform the study. Bias are effectively alleviated as for both in vivo and in vitro studies, the robust phenotype of our results is based on objective measurements instead of any human estimation.

Reporting for specific materials, systems and methods

We require information from authors about some types of materials, experimental systems and methods used in many studies. Here, indicate whether each material, system or method listed is relevant to your study. If you are not sure if a list item applies to your research, read the appropriate section before selecting a response.

Materials & experimental systems

n/a	Involved in the study
[ ]	[x] Antibodies
[ ]	[x] Eukaryotic cell lines
[x]	[ ] Palaeontology
[ ]	[x] Animals and other organisms
[x]	[ ] Human research participants
[x]	[ ] Clinical data

Methods

n/a	Involved in the study
[x]	[ ] ChIP-seq
[ ]	[x] Flow cytometry
[x]	[ ] MRI-based neuroimaging

Antibodies

Antibodies used	CyTOF antibodies are listed in supplementary table 1. All other antibodies used in the study are described in supplemental table 2.
Validation	All antibodies used are commercially available and validated by the manufacturers.

FITC anti-human CD4 Antibody, clone RPA-T4 (BioLegend 300506): https://www.biolegend.com/en-us/products/fitc-anti-human-cd4-antibody-825
PE anti-human CD8a Antibody, clone HIT8a (BioLegend 300908): https://www.biolegend.com/en-us/products/pe-anti-human-cd8a-antibody-762
APC anti-human CD279 (PD-1) Antibody, clone EH12.2H7 (BioLegend 329908): https://www.biolegend.com/en-us/products/apc-anti-human-cd279-pd-1-antibody-4413
PE anti-human CD366 (Tim-3) Antibody, clone F38-2E2 (BioLegend 345006): https://www.biolegend.com/en-us/products/pe-anti-human-cd366-tim-3-antibody-6121?GroupID=GROUP28
eFluor 660 anti-human Galectin 9 Monoclonal Antibody, clone 9M1-3 (Thermo Fisher Scientific 50-9116-42): https://www.thermofisher.com/antibody/product/Galectin-9-Antibody-clone-9M1-3-Monoclonal/50-9116-42
FITC anti-human CD3 Antibody, clone HIT3a (BioLegend 300306): https://www.biolegend.com/en-gb/global-elements/pdf-popup/fitc-anti-human-cd3-antibody-751?GroupID=GROUP28
PE anti-human CD14 Antibody, clone 63D3 (BioLegend 367104): https://www.biolegend.com/en-us/products/pe-anti-human-cd14-antibody-12011
FoxP3 Antibody, anti-human, APC, REAfinity (Miltenyi Biotec 130-125-580): https://www.miltenyibiotec.com/US-en/products/foxp3-antibody-anti-human-reafinity-rea1253.html#gref
Anti-mouse CD3-APC/Cy7 (BioLegend 100221): https://www.biolegend.com/en-us/products/apc-cyanine7-anti-mouse-cd3-antibody-6068
Anti-mouse CD8-APC (BioLegend 100711): https://www.biolegend.com/en-us/products/apc-anti-mouse-cd8a-antibody-150
Anti-mouse CD4-FITC (BD Biosciences 553046): https://www.bdbiosciences.com/us/applications/research/t-cell-immunology/th-1-cells/surface-markers/mouse/fitc-rat-anti-mouse-cd4-rm4-5-also-known-as-rm45/p/553046
Anti-mouse FoxP3-PE (Invitrogen 12-5773-80): https://www.thermofisher.com/antibody/product/FOXP3-Antibody-clone-FJK-16s-Monoclonal/12-5773-80
LEAF™ Purified anti-human CD3 antibody, clone OKT3 (BioLegend 317304): https://www.biolegend.com/en-us/products/ultra-leaf-purified-anti-human-cd3-antibody-7745?GroupID=GROUP28
LEAF™ Purified anti-human CD28 antibody, clone CD28.2 (BioLegend 302914): https://www.biolegend.com/en-us/products/purified-anti-human-cd28-antibody-632?GroupID=GROUP28
Purified anti-mouse Galectin-9 Antibody, clone 108A2 (BioLegend 137901): https://www.biolegend.com/en-gb/products/purified-anti-mouse-galectin-9-antibody-6562
Purified Rat Anti-Mouse CD16/CD32 (Mouse BD Fc Block™), Clone 2.4G2 (BD Biosciences 553142): https://www.bdbiosciences.com/us/applications/research/b-cell-research/surface-markers/mouse/purified-rat-anti-mouse-cd16cd32-mouse-bd-fc-block-24g2/p/553142
Human TruStain FcX™ (Fc Receptor Blocking Solution) (BioLegend 422302): https://www.biolegend.com/en-us/products/human-trustain-fcx-fc-receptor-blocking-solution-6462?GroupID=GROUP22
PD-1 (D4W2J) XP® Rabbit mAb (Cell Signaling Technology 86163): https://www.cellsignal.com/products/primary-antibodies/pd-1-d4w2j-xp-rabbit-mab/86163
Mouse anti-GALECTIN-9 Antibody, clone OTI1G3 (Bio-Rad VMA00212): https://www.bio-rad-antibodies.com/static/datasheets/vma00/human-galectin-9-antibody-oti1g3-vma00212.pdf
Peroxidase AffiniPure Goat Anti-Human IgG, Fcγ fragment specific (Jackson ImmunoResearch Inc. 109-035-098): https://www.jacksonimmuno.com/catalog/products/109-035-098
Peroxidase-AffiniPure Goat Anti-Rabbit IgG (H+L) antibody (Jackson ImmunoResearch Inc. 111-035-003): https://www.jacksonimmuno.com/catalog/products/111-035-003
Peroxidase-AffiniPure Goat Anti-Mouse IgG (H + L) antibody (Jackson ImmunoResearch Inc. 115-035-003): https://www.jacksonimmuno.com/catalog/products/115-035-003
Anti-mouse Galectin-9 antibody InVivoMAb, clone RG9-1 (Bio X Cell BE0218): https://bxcell.com/product/m-galectin-9/
Anti-TIM-3 Rabbit antibody (R&D Systems MAB23652): https://www.rndsystems.com/products/human-tim-3-antibody-2321c_mab23652
Anti-mouse GITR antibody InVivoMab, clone DTA-1 (Bio X Cell BE0063): https://bxcell.com/product/m-gitr/
Rabbit Anti-Stat1, phospho (Tyr701) Monoclonal Antibody, Unconjugated, Clone 58D6 (Cell Signaling Technology 9167): https://www.cellsignal.com/products/primary-antibodies/phospho-stat1-tyr701-58d6-rabbit-mab/9167
Rat IgG2b isotype control antibody InVivoMab (Bio X Cell BE0090): https://bxcell.com/product/rat-igg2b-isotype-control/
InVivoMAb anti-mouse PD-L1 (B7-H1) (Bio X Cell BE0101): https://bxcell.com/product/m-pdl-1/
Anti-FLAG M2 Magnetic Beads (Sigma-Aldrich M8823): https://www.sigmaaldrich.com/catalog/product/sigma/m8823?lang=en&region=US
Anti-FLAG M2 Affinity Gel (Sigma-Aldrich A2220): https://www.sigmaaldrich.com/catalog/product/sigma/a2220?lang=en&region=US
Dynabeads Pan Mouse IgG (Thermo Fisher Scientific 11041): https://www.thermofisher.com/order/catalog/product/11041#/11041
Mouse IgG2a, κ Isotype Ctrl LEAF™ Purified (BioLegend 400224): https://www.biolegend.com/en-us/products/purified-mouse-igg2a-kappa-isotype-ctrl-2622
Mouse IgG1, kappa Isotype Ctrl PE (BioLegend 400112): https://www.biolegend.com/en-us/products/pe-mouse-igg1-kappa-isotype-ctrl-1408?GroupID=GROUP29
Mouse IgG1, κ Isotype Ctrl Antibody APC (BioLegend 400120): https://www.biolegend.com/en-us/products/apc-mouse-igg1-kappa-isotype-ctrl-1404?GroupID=GROUP29

Eukaryotic cell lines

Policy information about cell lines

Cell line source(s)	The following cell lines were obtained from the American Type Culture Collection (ATCC): Jurkat (clone E6.1), EMT6, 293T, HeLa, Hep3B, T47D, MCF7, BT549, MB231, A549, H1975, THP-1, A375, HepG2, Tong/HCC, PC-3, DU-145, Miapaca-2, L3.6PL, H1650, HCC827, H441, H1229, SUM149, HCC1806, Py8119.<br>MC-38 was obtained from National Cancer Institute.
Authentication	Cells lines were authenticated by short tandem repeat DNA finger printing.
Mycoplasma contamination	Cell lines used in this study were tested negative for mycoplasma.
Commonly misidentified lines<br>(See ICLAC register)	No commonly misidentified cell lines were used.

Animals and other organisms

Policy information about studies involving animals; ARRIVE guidelines recommended for reporting animal research

Laboratory animals	C56BL/6J and BALB/cJ, female mice 6-8 weeks of age were used in the study.
Wild animals	This study did not involve wild animals.
Field-collected samples	This study did not involve field-collected samples.
Ethics oversight	All animal experiments were performed in accordance with The University of Texas MD Anderson Cancer Center (MDACC) Institutional Animal Care and Use Committee (IACUC) guidelines in an MDACC AAALAC accredited barrier facility vivarium.

Note that full information on the approval of the study protocol must also be provided in the manuscript.

Flow Cytometry

Plots

Confirm that:

- [x] The axis labels state the marker and fluorochrome used (e.g. CD4-FITC).
- [x] The axis scales are clearly visible. Include numbers along axes only for bottom left plot of group (a 'group' is an analysis of identical markers).
- [x] All plots are contour plots with outliers or pseudocolor plots.
- [x] A numerical value for number of cells or percentage (with statistics) is provided.

Methodology

Sample preparation	Samples used in flow cytometry were cell lines in culture, human PBMCs from a commercial source, or CD8 T cells isolated from human PBMCs using the EasySep™ Human CD8+ T Cell Isolation Kit from STEMCELL Technologies Inc.
Instrument	The data was collected on a BD FACSCanto II flow cytometer.
Software	All flow data was collected using FACSDIVA 8.0.1 (BD) and analyzed using FlowJo version 10.6.2 (BD).
Cell population abundance	CD8 T cells were isolated from human PBMCs by negative selection using the EasySep Human CD8+ T cell Isolation kit (StemCell Technologies) per the manufacturer's instructions. The CD8 T cell (CD3+CD8+) content of the isolated fraction is routinely >85%.
Gating strategy	Gating strategy is exemplified in Figure 4a and Supplementary Figure 7b, d, with gates drawn based on single-stain and full-minus-one (FMO) controls.

- [x] Tick this box to confirm that a figure exemplifying the gating strategy is provided in the Supplementary Information.
